# Dynamic labelling of neural connections in multiple colours by trans-synaptic fluorescence complementation

**DOI:** 10.1038/ncomms10024

**Published:** 2015-12-04

**Authors:** Lindsey J. Macpherson, Emanuela E. Zaharieva, Patrick J. Kearney, Michael H. Alpert, Tzu-Yang Lin, Zeynep Turan, Chi-Hon Lee, Marco Gallio

**Affiliations:** 1 Howard Hughes Medical Institute and Department of Biochemistry and Molecular Biophysics, Columbia University, New York, New York 10032, USA; 2 Department of Neurobiology, Northwestern University, Evanston, Illinois 60208, USA; 3 Section on Neuronal Connectivity, Laboratory of Gene Regulation and Development, Eunice Kennedy Shriver National Institute of Child Health and Human Development, National Institutes of Health, Bethesda, Maryland 20892, USA

## Abstract

Determining the pattern of activity of individual connections within a neural circuit could provide insights into the computational processes that underlie brain function. Here, we develop new strategies to label active synapses by trans-synaptic fluorescence complementation in *Drosophila*. First, we demonstrate that a synaptobrevin-GRASP chimera functions as a powerful activity-dependent marker for synapses *in vivo*. Next, we create cyan and yellow variants, achieving activity-dependent, multi-colour fluorescence reconstitution across synapses (X-RASP). Our system allows for the first time retrospective labelling of synapses (rather than whole neurons) based on their activity, in multiple colours, in the same animal. As individual synapses often act as computational units in the brain, our method will promote the design of experiments that are not possible using existing techniques. Moreover, our strategies are easily adaptable to circuit mapping in any genetic system.

## Abstract (web summary)

The ability to map the activity of synapses within a circuit will help in elucidating the neural basis of behaviour. Here, Macpherson *et al*. report new strategies to specifically label active synapses in Drosophila with multi-colour fluorescence tags.

## Graphical abstract

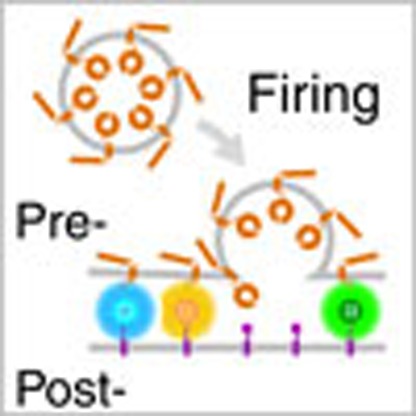

Mapping the connectivity of neural circuits is a key step towards a better understanding of the functional organization of the brain. Traditional techniques for mapping synaptic connections (such as paired recordings and electron microscopy) are powerful, yet they are difficult to apply on a large scale^1^,^2^. In addition, we still lack tools to mark specific connections based on their activity, or to co-label multiple synapses in the same animal. To overcome these challenges, we developed new strategies to trans-synaptically label the connections between genetically defined neuronal populations based on GFP (green fluorescent protein) Reconstitution Across Synaptic Partners (GRASP^3^), and utilized the fruitfly *Drosophila* to demonstrate their use *in vivo*. GRASP uses complementary split-GFP fragments (sp-GFP1–10 and sp-GFP11) expressed on the extracellular membranes of different neurons to reconstitute a functional fluorescent GFP reporter at the points of contact. GRASP was originally developed in *Caenorhabditis elegans* (*C. elegans*)^3^, and has been widely used for circuit mapping in *Drosophila* and mouse^4^,^5^,^6^,^7^,^8^. Here, we demonstrate two new modifications of GRASP: activity-dependent, synaptic GRASP and multi-colour X-RASP, which will expand the utility of GRASP for circuit mapping.

## Results

**Figure 1:**
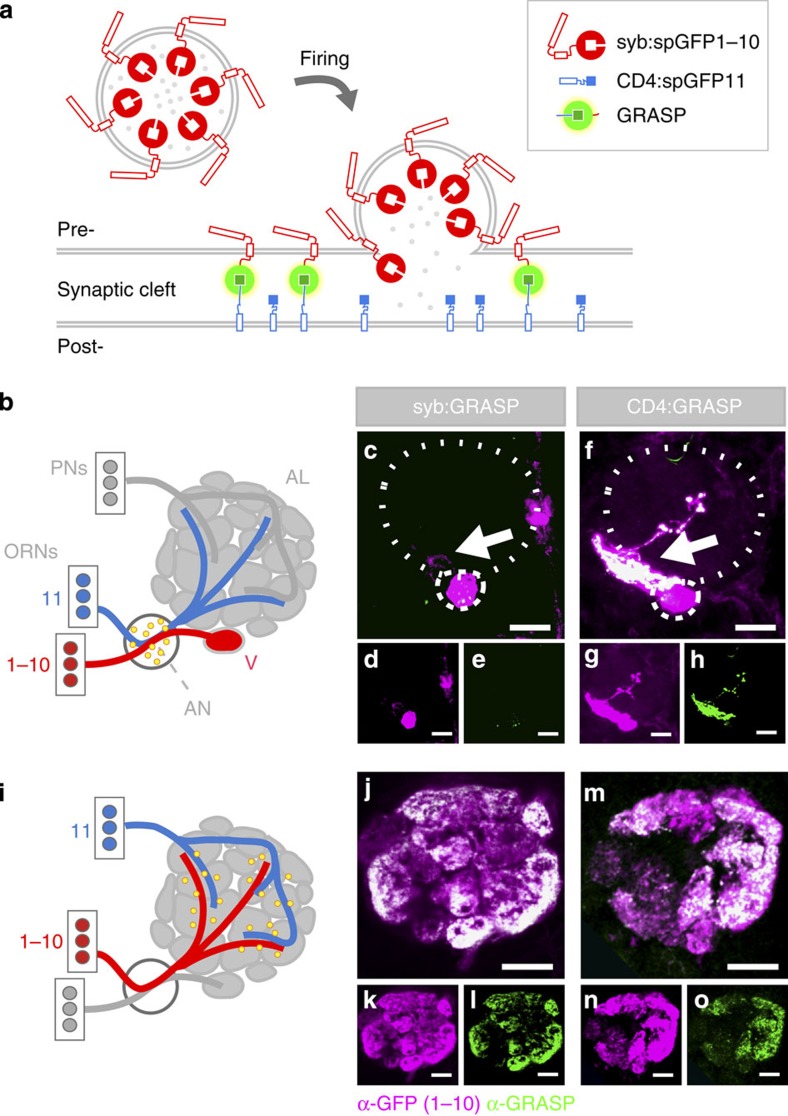
Design and validation of activity dependent synaptic GRASP in the *Drosophila* olfactory system. (**a**) Design of activity-dependent syb:GRASP. The spGFP1–10 molecule is sequestered to the lumen of synaptic vesicles by fusion to n-syb. During synaptic transmission, vesicles fuse to the presynaptic membrane, exposing spGFP1–10 to the synaptic cleft, and to reconstitution with spGFP11. (**b**–**o**) Validation of the synaptic localization of syb:GRASP. (**b**,**i**) Experimental design: the drawings represent the antennal lobe and the various cell types that innervate it (AL, antennal lobe; AN, antennal nerve; ORNs, olfactory-receptor neurons; PNs, projection neurons; V, V-glomerulus). The experiment summarized in **b** was designed to reveal potential extra-synaptic GRASP. (**c**–**e**) Extra-synaptic GRASP is virtually absent in flies expressing syb:spGFP1–10 in *GR21*-ORNs (targeting the V-glomerulus) in combination with spGFP11 in *Orco*-ORNs, which fasciculate together with *GR21a*-ORNs but do not form synapses with them. (**f**–**h**) However, CD4:spGFP1–10 produces significant extra-synaptic GRASP in the same conditions (mostly observed within the AN: arrows in **c**,**f**). The experiment summarized in **i** is designed to achieve appropriate trans-synaptic GRASP in the AL as a control. Indeed, expressing either (**j**–**l**) syb:spGFP1–10 or (**m**–**o**) CD4:spGFP1–10 in ORNs (*Orco* promoter) produces robust GRASP (**l**,**o**) in combination with spGFP11 expressed in post-synaptic projection neurons (PNs, *GH146* promoter). (**c**–**o**) Confocal projections of a single AL stained with an anti-GFP1–10 (magenta) and anti-GRASP (green, seen as white due to overlap in **c**,**f**,**j**,**m**). (**d**,**g**,**k**,**n**) Magenta channel. (**e**,**h**,**l**,**o**) Green channel. Scale bar, 20 μm. Full genotypes: (**c**) *UAS-syb:spGFP1–10/Aop-CD4:spGFP11; Gr21a-Gal4/Orco-LexA*; (**d**) *UAS-CD4:spGFP1–10/Aop-CD4:spGFP11; Gr21a-Gal4/Orco-LexA;* (**f**) *Orco-Gal4/GH146-LexA, Aop-CD4:spGFP11; UAS-syb:spGFP1–10/+;* (**g**) *Aop-CD4:spGFP1–10, UAS-CD4:spGFP11/GH146-Gal4; Orco-LexA/+*.

In existing GRASP methods, GFP reconstitution can in principle occur at any time neurons come into contact during development or synapse formation, resulting in signals from contacts other than active synapses. Therefore, as a first step towards establishing new, dynamic GRASP tools for use in flies, we engineered a Neuronal-synaptobrevin:spGFP1–10 chimera (syb:spGFP1–10) for synaptic GRASP^9^,^10^. N-synaptobrevin (n-syb) is a key constituent of the synaptic vesicle membrane and has been previously used as a carrier to achieve synaptic localization of GFP^11^,^12^. As expected, our syb:spGFP1–10 chimera was effectively trafficked to synapses, demonstrating good colocalization with the presynaptic marker Bruchpilot^13^ (Supplementary Fig. 1). Furthermore, in our design spGFP1–10 is fused to the C terminus of *Drosophila* n-syb, and would be exposed to the synaptic cleft (and therefore available for reconstitution with spGFP11) only after vesicle fusion (Fig. 1a). As fusion events are triggered by calcium entry into the presynaptic terminal during an action potential, we reasoned that neuronal firing would result in a dramatic increase in the availability of spGFP1–10 for binding, and therefore in preferential labelling of active synapses, that is, producing ‘activity-dependent' GRASP.

### Validating the synaptic specificity of syb:GRASP

GRASP is generally used as a static marker for synaptic connections. Synaptic GRASP has been demonstrated in *C. elegans*^3^, mouse^7^,^8^ and *Drosophila*^4^, and our syb:GRASP fusion has been recently used to map connections in the visual and thermosensory systems^9^,^10^ (see extended data Fig. 2 in Frank *et al*.^9^, and Fig. 3 in Karuppudurai *et al*.^10^). Our first goal here was to more rigorously test the synaptic specificity of the syb:GRASP system *in vivo*, and for this, we utilized the fly olfactory system as a powerful and well-characterized genetic platform^14^. We expressed syb:spGFP1–10 in a single population of olfactory-receptor neurons (ORNs, under the control of *GR21a-Gal4* (refs 15, 16)) and its GRASP partner CD4:spGFP11 in a large, non-overlapping group of ORNs (expressing the driver *Orco-LexA*^17^, Fig. 1b–e). As different classes of ORNs fasciculate together extensively in the antennal nerve, but do not form synaptic connections with each other^14^, this experiment was designed to reveal potential signals from non-synaptic contacts. As shown in Fig. 1c, syb-targeted spGFP1–10 produced little or no extra-synaptic GRASP. In contrast, spurious GRASP signal was observed within the antennal nerve in animals with the same genetic constitution but expressing the broadly localized CD4:spGFP1–10 construct (Fig. 1f). Both syb:spGFP1–10 and CD4:spGFP1–10 provide robust signals when matched with CD4:spGFP11 expressed in the appropriate post-synaptic cells (that is, ‘second-order' neurons of the olfactory system or projection neurons (PNs)^14^, Fig. 1i–o, and see Supplementary Fig. 2 for validation of the specificity of the antibodies used in this study).

**Figure 2:**
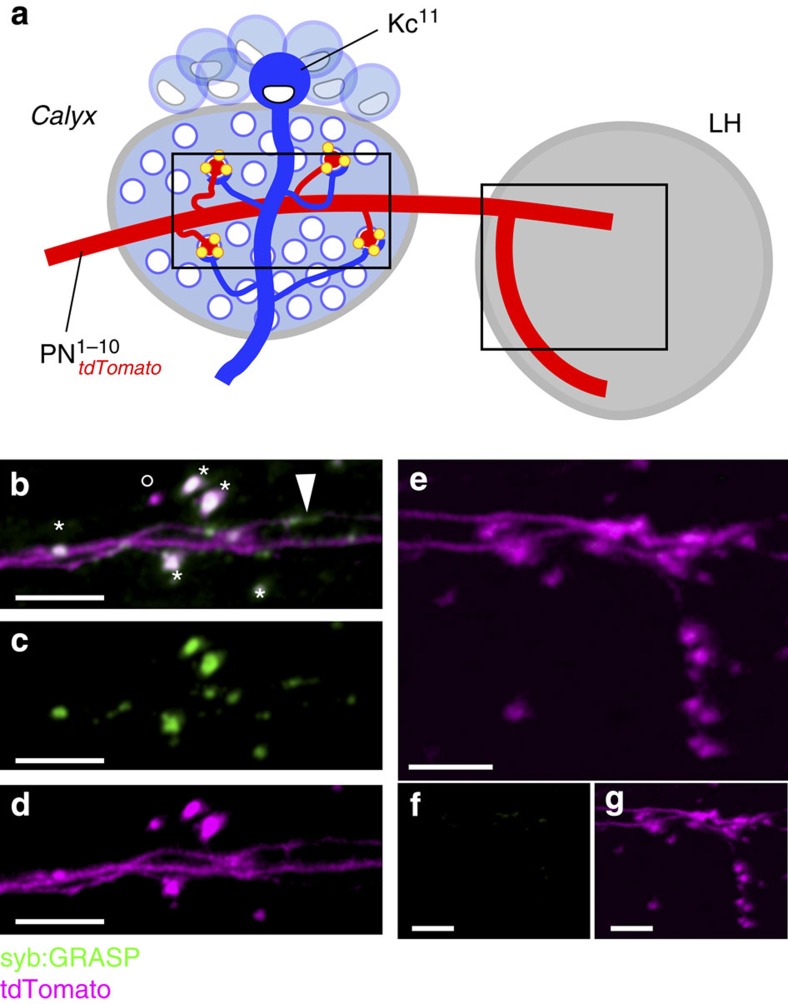
syb:GRASP labelling in the mushroom body calyx. (**a**) Experimental set-up: to visualize the syb:GRASP-labelled synapses of individual neurons, the driver *R68D02-LexA* was used to express syb:spGFP1–10 in two to three olfactory PNs (also labelled by tdTomato, red), while CD4:spGFP11 was targeted to Kenyon cells of the mushroom body (Kc, blue; by MB247-Gal4). Olfactory PNs synapse onto Kenyon cell dendrites in the mushroom body calyx, and then continue to the lateral horn (LH) where they also form synapses. (**b**–**d**) Robust syb:GRASP fluorescence is detected by live two-photon microscopy in the mushroom body calyx (asterisks). Some non-synaptic GRASP is also observed along the axon (arrowhead), and at least one potential synaptic contact is not labelled by GRASP fluorescence (open circle). (**e**–**g**) As expected, no syb:GRASP signal is observed in the LH, the secondary site of PN synapses, since spGFP11 is not expressed in the LH neurons. (**b**,**e**) Composite image. (**c**,**f**) Green channel, GRASP. (**d**,**g**) Magenta channel, tdTomato. Scale bar, 5 μm. Full genotype: *Aop-tdTomato/+; Aop-syb-spGFP1–10, UAS-CD4:spGFP11/R68D02-LexA; MB247-Gal4/+*.

For an additional test of specificity, we targeted the next-order synapse of the olfactory system (that is, between olfactory PNs and Kenyon cells of the mushroom body^14^). We expressed the syb:spGFP1–10 construct in two to three olfactory PNs (under the control of *R68D02-LexA*, also driving the red fluorescent protein tdTomato, Fig. 2), while CD4:spGFP11 was driven by a promoter active in a large subset of Kenyon cells (*MB247-Gal4* (refs 18, 19)). In this set-up, the axons of the PNs expressing syb:spGFP1–10 traverse the Calyx, a dense neuropil comprised of the dendrites of ∼2,000 Kenyon cells expressing the broadly localized CD4:spGFP11, before proceeding to terminate within the lateral horn (LH; Fig. 2a). Hence, synaptic GRASP should be observed between each PN terminal within the Calyx and Kenyon cell dendrites, while no signal is expected within the LH. Two-colour, two-photon microscopy demonstrated that indeed GFP reconstitution was mainly localized to PN boutons within the Calyx (asterisks in Fig. 2b), with limited occurrence of extra-synaptic signal (arrowhead in Fig. 2b); in rare cases, boutons could be observed lacking GRASP signal (empty circle in Fig. 2b; 3 boutons of 67, in 14 preparations examined). No GFP reconstitution was observed within the LH (Fig. 2e; see also Frank *et al*.^9^, and Karuppudurai *et al*.^10^ for additional control experiments in the thermosensory and visual systems, respectively).

### Activity-dependent GFP reconstitution at synapses

**Figure 3:**
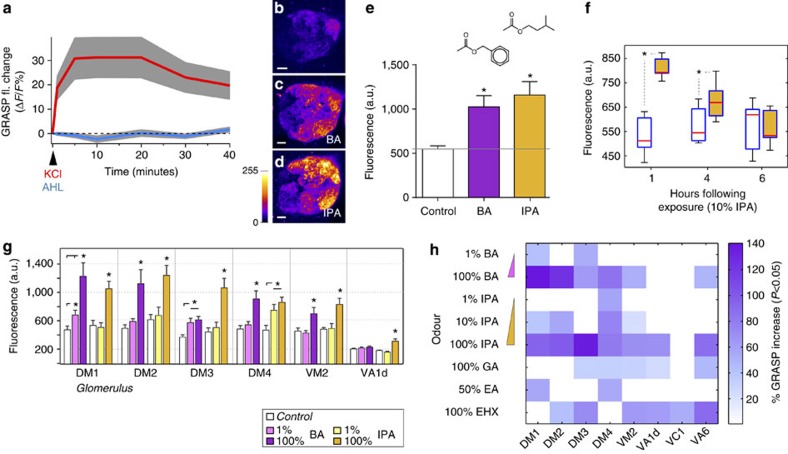
Activity-dependent labelling of olfactory synapses by syb:GRASP. (**a**) Brief exposure to 70 mM KCl rapidly increases syb:GRASP fluorescence at the ORN–PN synapse of the olfactory system. Isolated brains were exposed to KCl and imaged under live, time-lapse microscopy. Each point represents the fluorescence measured across the entire antennal lobe (AL, from a maximum projection) and plotted as fluorescence change from *t*=0 (as Δ*F*/*F*%). Controls (blue line) were exposed to the same treatment but omitting KCl (AHL, adult haemolymph; *n*=7 brains for KCl, *n*=6 for AHL. Grey regions ±s.e.m.). (**b**–**e**) Exposure to isopentyl acetate (IPA, banana smell) or benzyl acetate (BA, jasmine smell) significantly increases syb:GRASP at the ORN–PN synapse. (**b**–**d**) Representative AL stack projections of control, BA- and IPA-exposed flies. Scale bar, 10 μm. (**e**) Quantification of the average intensity of GRASP fluorescence across the AL (*n*=8 for control, *n*=4 for IPA, *n*=5 for BA, *P<0.05 using one-tailed *t*-tests; error bars=s.e.m.; a.u.s, arbitrary fluorescent units). (**f**) Time course of GRASP signal decay after exposure to 10% IPA measured across the AL (for 1 h, *n*=7 for control, *n*=6 for IPA; for 4 h *n*=5 for control, *n*=8 for IPA; for 6 h, *n*=6 for control, *n*=6 for IPA, *P<0.05 using one-tailed *t*-tests; the edges of the boxes are the first and third quartiles, and red lines marked the medians, whiskers=data range; a.u.=arbitrary fluorescence units). (**g**) syb:GRASP fluorescence in individual AL glomeruli in response to presentations of diluted (1%) and undiluted (100%) BA (pink bars) and IPA (yellow bars). Different glomeruli respond to the two odours with different affinities (*P<0.05; one-tailed *t*-test, *n*=6–10; error bars=s.e.m.). (**h**) syb:GRASP glomerular responses to a small battery of odours and odour concentrations. As expected, different patterns of glomerular activation are recorded in both an odour-dependent and concentration-dependent manner (the colour of each box represent the % GRASP fluorescence increase compared with the appropriate control, only significant changes are plotted; *P*<0.05; one-tailed *t*-test, *n*=5–10; additional odours used: EA, ethyl acetate; EHX, ethyl hexanoate; GA, geranyl acetate). Full genotype: *Orco-Gal4/GH146-LexA, Aop-CD4:spGFP11; UAS-syb:spGFP1–10/+*.

Next, we tested if the syb:GRASP system is capable of preferential labelling of active synapses. We again used flies in which trans-synaptic GFP reconstitution was targeted to the first central synapse of the olfactory system (ORN–PN synapse). To achieve robust, simultaneous activation of the synapses of interest and follow the development of the GRASP signal by live time-lapse microscopy, we developed an ‘*ex vivo*' preparation in which a freshly isolated live brain is exposed to a rapid depolarizing drive by brief application of KCl (3 × 5 s). Using two-photon microscopy, we then acquired full stacks of the antennal lobe (AL) at 1–10 min intervals, and processed the images to extract quantitative data. Our results show an activity-dependent boost of the syb:GRASP signal at 5 min after treatment, and persisting for at least 40 min (Fig. 3a; and note that we did not observe a significant boost in signal when using CD4:GRASP). The fact that the boost in syb:GRASP was already measurable after as little as 5 min suggests that it is likely a result of activity-dependent vesicle fusion events rather than, for example, of new synaptogenesis.

### A strategy for retrospective labelling of active synapses

The above ‘*ex vivo*' experiment demonstrates that n-syb targeting confers activity-dependent properties to trans-synaptic GFP reconstitution, resulting in a boost of GRASP fluorescence that lasts well after activity is gone. We reasoned that the persistence of the syb:GRASP signal could afford a unique way to retrospectively mark active synapses *in vivo*. To test this, we again used strains expressing syb:GRASP at the ORN–PN synapse, and performed imaging experiments on flies that had been either grown on an odour-free sugar–agarose substrate or, alternatively, exposed to olfactory stimuli for 4 × 10 min followed by up to 6 h of ‘rest' (see Methods for details). Compared with the control fly cohort, exposure to strong olfactory cues resulted in an increase in syb:GRASP fluorescence at the ORN–PN synapse by 80–110% (Fig. 3b–e)—reflecting an odour-dependent activity pattern experienced by the fly nearly 1 h before. Even when using a lower odour concentration (10% isopentyl acetate—resulting in a ∼60% boost at 1 h, Fig. 3f), this increase in fluorescence persisted for up to 4 h after exposure, providing an ample window of opportunity for retrospective labelling.

We also used the ORN-PN synapse to test both the sensitivity (by using low odour concentrations) and specificity of the syb:GRASP responses. As expected, low odour concentrations activated fewer glomeruli, while increasing odour concentrations resulted in a dose-dependent boost in fluorescence for some glomeruli, while others showed no further increase, and yet others only responded to high concentrations (Fig. 3g). This likely results from different OR affinities for the odour. Moreover, a small battery of odours and odour concentrations produced different patterns of glomerular activation in both an odour-dependent and concentration-dependent manner (Fig. 3h), in general good agreement with glomerular patterns recorded acutely in live imaging studies^20^,^21^,^22^. Interestingly, the dynamic range of GRASP responses for each glomerulus correlated well with the dynamic range of firing for the ORN type that innervates it (as compiled from literature, see Supplementary Table 1 and Supplementary Fig. 3); a notable exception was the DM1 glomerulus, characterized by a particularly large syb:GRASP dynamic range.

### Labelling of active thermosensory and visual synapses

**Figure 4:**
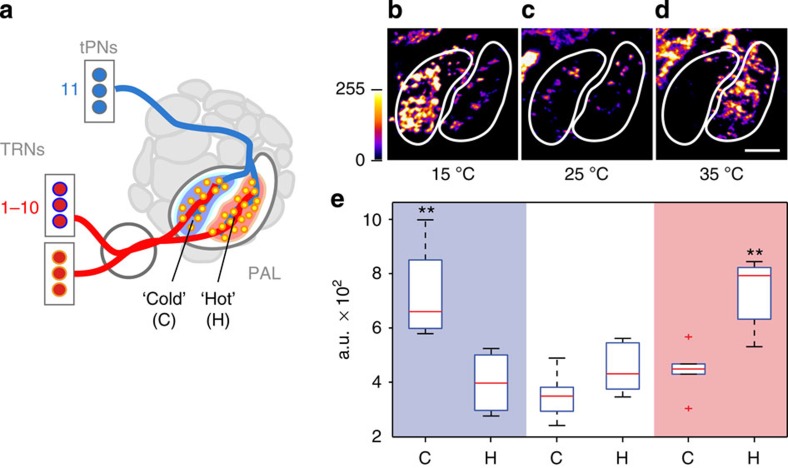
Activity-dependent GRASP in the thermosensory circuit. (**a**) Experimental set-up: syb:spGFP1–10 is expressed in both hot and cold temperature-receptor neurons (TRNs, using *Ir93a-LexA*), while spGFP11 is active in a unique group of ‘broadly tuned' thermosensory-projection neurons (tPNs, that is, activated by either heating or cooling; by *VT40053-Gal4*). TRNs responding to either cold or hot stimuli synapse within distinct regions of the posterior antennal lobe (PAL), the cold and hot glomeruli (C and H). (**b**–**e**) Brief exposure to either cold (10 min at 15 °C) or hot (35 °C) temperatures increases syb:GRASP fluorescence in the expected glomerulus (control flies were kept at 25 °C). (**b**–**d**) Representative PAL stack projections of flies exposed to cold (15 °C), control (25 °C) and hot (35 °C). Scale bar, 20 μm. While **e** shows quantitation of the average fluorescence intensity of syb:GRASP within hot and cold glomeruli (the edges of the boxes are the first and third quartiles, and red lines marked the medians, whiskers=data range. Outliers are displayed as red crosses; *n*=6 for control (25 °C), *n*=4 for cold (15 °C), *n*=6 for hot (35 °C); **P<0.005 using two-tailed *t*-test; a.u.=arbitrary fluorescence units). Full genotype: *w; Aop-syb:spGFP1–10; UAS-CD4:spGFP11/+; IR93a-LexA/VT40053-Gal4*.

To further test the ability of our system to retrospectively report specific patterns of synaptic activation *in vivo*, we turned to the thermosensory system. In *Drosophila*, rapid temperature changes are detected by dedicated hot and cold temperature-receptor neurons (TRNs) in the last antennal segment, the arista^23^. The projections of these sensory neurons innervate the base of the AL, where hot and cold TRNs form two distinct, adjacent glomeruli defining a simple map for temperature representation (Fig. 4a; ref. 23). For this experiment, we expressed syb:spGFP1–10 in both hot and cold TRNs (using *IR93a-LexA*^9^), while CD4:spGFP11 was directed to a subset of their targets: a unique population of ‘broadly tuned' thermosensory-projection neurons (tPNs) that respond to both heating and cooling and are required for behavioural aversion to both hot and cold temperature ranges (labelled by *VT40053-Gal4*^9^, Fig. 4a). First, we confirmed that the targeting of syb:GRASP at this synapse had no effect on the flies' temperature preference behaviour in our rapid two-choice assay^23^ (Supplementary Fig. 4). Next, we exposed flies to a brief temperature shift from 25 °C to either 15 °C or 35 °C (limited to 10 min to minimize the effects of temperature on Gal4 and LexA-mediated gene expression), following which we quantified changes in fluorescence at the TRN–tPN synapse. As expected, we observed a significant boost in syb:GRASP in the ‘cold' glomerulus (that is, the region in which cold sensing TRNs contact *VT40053* tPNs) in flies exposed to cold temperatures, while exposure to hot temperatures resulted in a similar fluorescence increase in the ‘hot' glomerulus (that is, the region in which hot-sensing TRNs contact *VT40053* tPNs, Fig. 4b–e). Hence, by looking at the levels of syb:GRASP in hot and cold glomeruli, we could in principle infer the temperature range that the fly was exposed to before the end of the experiment.

Finally, we also observed an activity-dependent syb:GRASP boost when we targeted our reagents to the next-order synapse of the olfactory system (that is, between PNs and Kenyon cells; Supplementary Fig. 5a–c), or to the visual system (Supplementary Fig. 5d–g, and see figure legends and Methods for details), illustrating application of this system to synapses that are not organized in glomerular structures. Taken together, our data demonstrate that syb:GRASP represents a unique way to retrospectively mark active synapses *in vivo*, recording patterns of activity as persistent signals that can be later visualized under a microscope.

### Multi-colour labelling of active synapses with X-RASP

**Figure 5:**
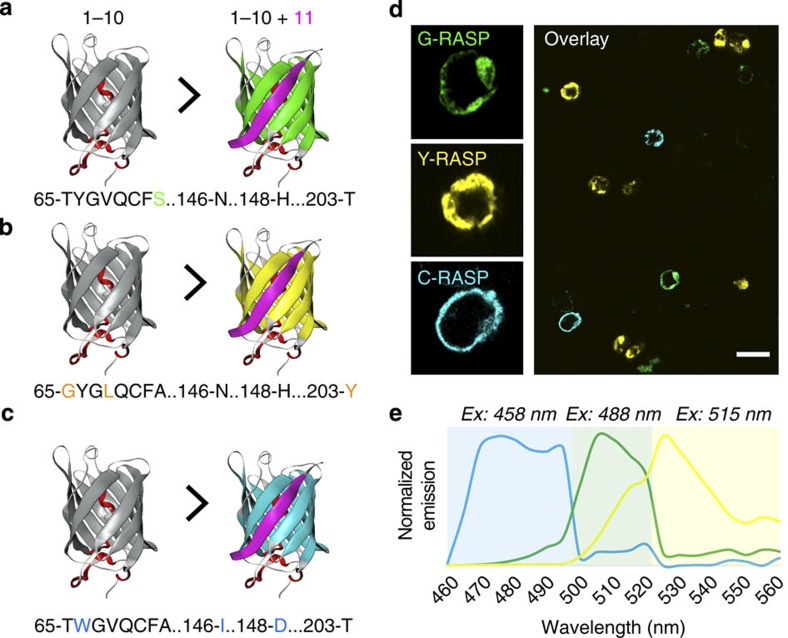
Multi-colour X-RASP *in vitro*. (**a**–**c**) Diagram depicting the mutations introduced in the spXFP1–10 fragment to obtain yellow and cerulean X-RASP when reconstituting fluorescence with the invariant spXFP11 (represented in pink). (**d**) *In vitro* X-RASP fluorescence in HEK293 cells. Each coloured X-RASP was produced by transfecting the syb:spGFP1–10, syb:spYFP1–10 or syb:spCFP1–10 with spXFP11 into separate wells. After 24 h of incubation to allow expression, the cells were mixed together, fixed and imaged by confocal microscopy. Left panels show magnified individual G-, Y- and C-RASP cells. Scale bar, 20 μm. (**e**) Normalized emission spectra measured by confocal microscopy using spectral detection settings (see Methods for details). C-RASP (blue trace), G-RASP (green trace) and Y-RASP (yellow trace) were obtained by co-transfection of HEK293 cells as in **d**.

To make our reagents more versatile, our next goal was to add the ability to label multiple synapses in the same animal with different colours. Since GRASP uses GFP as an indicator of synaptic contact, we introduced colour-shifting mutations in the GFP chromophore to alter the emission spectra of the reconstituted product. Indeed, the majority of the mutations that produce such shifts are located within beta barrels 1–10 of GFP^24^ (that is, spGFP1–10), so that reconstitution with the constant spXFP11 (where X stands for a given fluorescent protein) is expected to produce different X-RASP products. As a result, the synapses of neurons expressing each spXFP1–10 variant could be differentially labelled when converging on common targets expressing spXFP11. Yellow- and cerulean-FPs feature good spectral separation from GFP while maintaining comparable brightness^25^,^26^. We used syb:spGFP1–10 as a starting point, and introduced mutations to make yellow and cerulean versions using site-directed mutagenesis. For yellow, we introduced the amino-acid changes T65G, V68L, S72A and T203Y^26^, while for cerulean we used Y66W, S72A, N146I and H148D^25^ (Fig. 5a–c; and see Supplementary Fig. 6 for details). As expected, co-expression of the syb:spXFP1–10 colour variants with the constant spXFP11 (in HEK293 cultured cells) produced bright, appropriately colour shifted Y-RASP and C-RASP *in vitro* (Fig. 5d,e). Next, we tested these variants *in vivo*, in the fly olfactory system—to ensure faithful multi-colour labelling of synapses in the brain. To increase the flexibility of our transgenic tools, we wanted to allow for independent expression of each syb:spXFP1–10 variant in any given population of genetically defined neurons, while at the same time driving the invariant spXFP11 in their synaptic targets (that is, for trans-synaptic reconstitution). For this, we engineered flies expressing the split-XFP fragments under the control of three orthogonal expression systems: Gal4 (ref. 27), LexA^17^ and Q^28^ (see Supplementary Table 2 for a list of reagents created for this study). As the three systems do not interact, this strategy allows independent expression of the constructs in different combinations of pre- and post-synaptic cells.

**Figure 6:**
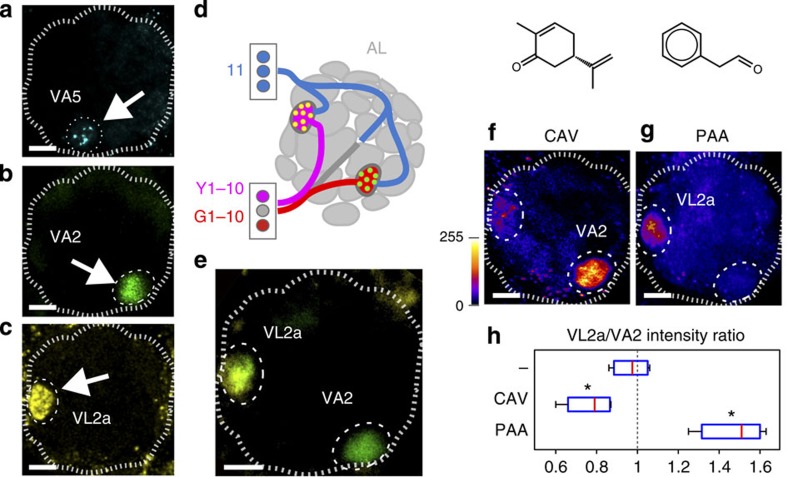
Multi-colour X-RASP labels active synapses *in vivo* in the fly olfactory system. (**a**) Cerulean, (**b**) green and (**c**) yellow X-RASP is observed in individual glomeruli of the AL when the corresponding syb:spXFP1–10 fragments are expressed by specific OR promoters and spXFP11 is broadly expressed in *GH146* PNs (common targets of ORNs). (**a**) C-RASP in VA5 (drivers: *Or49b-Q*, *GH146-LexA*), (**b**) G-RASP in VA2 (*Or92a-Gal4*, *GH146-Q*) and (**c**) Y-RASP in VL2a (*Ir84a-LexA*, *GH146-Gal4*). (**a**–**e**) Small confocal stacks (∼70 × 70 × 5 μm). (**d**) Experimental design for two-colour X-RASP in a single fly: distinct populations of ORNs express either syb:spYFP1–10 (driver: *IR84a-LexA*, active in ORNs innervating the glomerulus VL2a) or syb:spGFP1–10 (*Or92a-Gal4*, targeting VA2); spXFP11 is expressed in *GH146* PNs. (**e**) Green and yellow X-RASP is seen at the appropriate locations in the glomerular map. (**f**–**h**) Exposing these flies to either S-carvone (CAV, caraway seed odour, **f**)—which preferentially activates VA2, or phenylacetaldehyde (PAA, rose odour, **g**)—which preferentially activates VL2a, produces a boost in the fluorescence of the appropriate glomerulus. (**f**,**g**) Maximum projections of two-photon stacks representing the entire AL; a fire LUT was applied to emphasize differences in intensity. (**h**) Fluorescence intensity ratio of VL2a to VA2 at baseline (VL2a/VA2, no odour=dash), and after exposure to CAV and PAA. CAV preferentially activates VA2 resulting in a VL2a/VA2 ratio <1, conversely PAA preferentially activates VL2a resulting in a ratio >1 (*P<0.05 in two-tailed *t*-tests with no-odour control, *n*=4 per condition). In **a**–**c** and **e**–**g**, the AL and the relevant glomeruli are circled. Scale bar, 10 μm (in all panels). Full genotypes: (**a**) *Or49b-QF, QUAS-syb:spCFP1–10/x,y; GH146-LexA, Aop-CD4:spXFP11/+*; (**b**) *GH146-Q, QUAS-CD4:spXFP11/+; OR92a-Gal4, UAS-syb:spGFP1–10/+*; (**c**) *IR84a-LexA, Aop-syb:spYFP1–10/GH146-Q, QUAS-CD4:spXFP11*; (**e**–**g**) *IR84a-LexA, Aop-syb:spYFP1–10/GH146-Q, QUAS-CD4:spXFP11; OR92a-Gal4, UAS-syb:spGFP1–10/+*.

To test multi-colour X-RASP in the fly olfactory system, we first expressed the invariant CD4:spXFP11 in PNs (under the control of *GH146*, see above and Methods) and syb:spGFP1–10, syb:spYFP1–10 or syb:spCFP1–10 in different genetically defined classes of ORNs (using *Or92a*, *IR84a* and *Or49b* drivers, respectively^15^,^16^,^29^; Fig. 6a–c). Each class of ORNs converges onto a different glomerulus, but all these glomeruli are innervated by GH146-expressing PNs and as a result we observed green, yellow and cerulean reconstitution restricted to the appropriate location in the glomerular map (Fig. 6). We could also demonstrate *in vivo* colour coding of two types of ORN–PN synapses in the same animal by simultaneous targeting of yellow and green syb:spXFP1–10 to different ORNs, in combination with PN expression of CD4:spXFP11 (Fig. 6d,e). Two-colour syb:X-RASP flies also provided a simple platform in which to test the activity dependence of X-RASP *in vivo*: we exposed flies to an odour expected to activate preferentially the ‘green' glomerulus (S-carvone or ‘caraway seed' odour^20^, Fig. 6f) or, alternatively, the ‘yellow' one (phenylacetaldehyde or ‘rose' odour^29^, Fig. 6g), and quantified the G-RASP and Y-RASP signal at the two glomeruli. Figure 6f–h shows that exposure to each odour produced a relative boost in the X-RASP fluorescence at the appropriate glomerulus, again reflecting a brief exposure to one of the two odours that happened nearly 1  h before.

## Discussion

Here, we validate a transgenic toolbox for activity-dependent, multi-colour labelling of synaptic connections between genetically defined circuit elements in *Drosophila*. There are well-established strategies to statically label synaptic connections (GRASP^3^, STaR^30^ and so on) and to acutely study synaptic activity (synaptoPhluorin^12^ or GCaMP^31^ for example). There are also transgenic tools to persistently label neurons *in vivo* based on their activity (c-fos, arc^32^; Tango-Trace^33^; Calexa^34^, Campari^35^ and so on). Our system is the first one that allows retrospective labelling of *synapses* based on their activity, in multiple colours, in the same animal. As individual synapses often act as computational units in the brain^36^, we envision that our strategies could inspire experiments in which different colours are used to study rearrangements at groups of synapses that happen as a result of plasticity, or developmental changes in the connectivity rules between neurons labelled by different X-RASP variants. Moreover, our tools will make it possible to retrospectively label the synaptic connections that are activated during complex behaviour in naturalistic settings, as well as to study changes in activity that happen at a slower time scale, where acute physiology is difficult to apply. As our approaches are easily applicable to any genetic system, they will make it easier to mark, identify and distinguish active synapses from multiple cell types in the very same animal, both statically and dynamically.

## Methods

### Construction of transgenes

*syb:spGFP1–10*: the *Drosophila n-syb:spGFP1–10* fusion construct was made essentially as described by Estes *et al*. for *n-syb:GFP*^11^; see also Supplementary Fig. 6a). First, the n-syb fragment was amplified by PCR using primers spanning the EcoRI-XhoI restriction sites (XhoI removes the stop codon of n-syb, and allows cloning the downstream spGFP1–10 fragment in frame). Using the template spGFP1–10 plasmid (*ace-4 CD4-2 spGFP1–10* gift of C. Bargmann), primers amplifying just the *spGFP1–10* portion were used, introducing XhoI and XbaI restriction sites at the 5′ and 3′ ends of the product, respectively. The *n-syb* and *spGFP1–10* PCR products were cloned into PCR2.1 TOPO vectors (Life Technologies) and sequenced. Restriction fragments EcoRI-XhoI n-syb and XhoI-XbaI were isolated, and then ligated into the pUAST and pLOT vectors cut with EcoRI-XbaI to create *UAS-syb:spGFP1–10* and *Aop-syb:spGFP1–10*, respectively. To insert *CD4:spXFP11* into pUAST and pQUAST, the entire *pat3-spGFP11:CD4* fragment was amplified by PCR from the template *spGFP11* (*rig-3 CD4-2 spGFP11*, gift of C. Bargmann), using primers to introduce BglII and XbaI restriction sites. These PCR products were then digested, and ligated into pUAST and pQUAST, and then sequenced. These vectors were injected and transgenic lines were created with different insertion sites. These are listed in Supplementary Table 2. To construct *Or49b-QF*, primers spanning the promoter region of *Or49b* were PCR amplified from fly genomic DNA, and cloned into pCR8/GW/TOPO vector. After sequencing to confirm the correct sequence and orientation for gateway cloning, L/R clonase was used to shuttle the *Or49b* promoter into the pBP-QF(Dest) vector (gift of D. Hattori). This vector was injected using the attP18 landing site for incorporation into the X-chromosome.

### Mutagenesis

The Quik-Change II mutagenesis kit (Agilent, Inc.) was used to introduce point mutations in the *spGFP1–10* TOPO vector, using specific PCR primers. *spYFP1–10* and *spCFP1–10* were created in a series of 3–4 rounds of mutagenesis, followed by sequencing (see Supplementary Fig. 6b for an alignment). The cerulean Y145A mutation was not included to preserve the superfolder mutations necessary for reconstitution^37^). The colour variants were then introduced along with n-syb into pLOT or pQUAST using the same set of restriction enzymes as described above.

### Fly strains

All strains developed for this study are listed in Supplementary Table 2. *Aop-CD4:spGFP11* and *UAS-CD4:spGFP1–10* were gifts of K. Scott. Drivers *GH146-Gal4*, *GH146-LexA*, *GH146-QF*, *Orco-Gal4*, *Orco-LexA*, *Or92a-Gal4*, *GR21a-Gal4*, *VGlut-Gal4*, *MB247-Gal4* and *MB247-LexA* are available from the Bloomington Stock Center. *IR84a-LexA* was a gift from R. Benton. *panR8-Gal4* (ref. 38; a combination of *rh5-Gal4*+*rh6-Gal4*) and *Tm5c-LexA*^10^ have been previously published, as have *Ir93a-LexA*^9^ and *VT40053-Gal4* (ref. 9). Flies used for Fig. 1 came from regular laboratory fly vials, were not sexed and were 2–6 days old. Flies used in Fig. 2 were all females (due to constraints of the genetic cross), and 6–7 days old—this is because the tdTomato signal was difficult to detect in younger flies.

### Statistical criteria

Sample size was not pre-determined, no samples were excluded from the analysis, no randomization/blinding was applied.

### GRASP immunohistochemistry

Immunofluorescence of GRASP (Fig. 1) was performed on 4% PFA-fixed, whole mount brains as described in Gordon and Scott^6^. Antibodies: mouse anti-GFP (1:100; Sigma, cat #G6539, referred to as anti-GRASP^6^), chicken anti-GFP (1:1,000, Abcam #13970, that preferentially recognizes spGFP1–10—see Supplementary Fig. 2). Secondary antibodies were used at 1:1,000 dilution: Donkey anti-mouse and anti-chicken Alexa 488 and 549. Immunostained brains were imaged using an Olympus or Zeiss confocal microscope. For the visual system, Mouse 24B10 (DSHB, 1:50), and rabbit anti-hCD4 (1:500, Novus Biologicals # NBP1-86143) were used with secondary goat anti-mouse, and anti-rabbit antibodies conjugated with Alexa Fluor 568, or 647 (Life Technologies) at a 1:400 dilution. For NMJ staining, third instar larvae were dissected and fixed in 4% PFA, and stained using chicken anti-GFP (1:1,000, Abcam #13970), goat anti-HRP (1:500, Jackson Immuno 123-005-021), mouse monoclonal nc-82 (bruchhpilot, 1:200). Secondary antibodies were used at 1:1,000 dilution Donkey anti-mouse cy3, Donkey anti-Chicken Alexa 488, and donkey anti-goat Alexa 633 (Jackson Immunoresearch). For quantification of syb:spXFP1–10 expression (Supplementary Table 2), flies were stained with chicken anti-GFP (1:1,000, Abcam #13970) using secondary antibody Donkey anti-Chicken Cy3 (1:1,000 Jackson Immunoresearch). The ALs were imaged by confocal microscopy, and fluorescence intensity was quantified by averaging the AL stack on the *z*-axis, and measuring the mean pixel intensity of an region of interest (ROI) encompassing the entire AL.

### KCl induction of syb:GRASP

We crossed *Orco-Gal4/UAS-syb:spGFP1–10* with *GH146-LexA/Aop-CD4:spGFP11*, and isolated progeny pupae on sorbitol agarose substrate (see below). Within 24 h of eclosion, flies were anaesthetized on ice, and their brains were dissected in adult haemolymph (AHL containing 108 mM NaCl, 5 mM KCl, 4 mM NaHCO_3_, 1 mM NaH_2_PO_4_, 5 mM Trehalose, 10 mM Sucrose, 5 mM HEPES, 8.2 mM MgCl_2_, 2 mM CaCl_2_, pH 7.4), placed in a 35 × 10 mm petri dish and imaged by two-photon microscopy. For the KCl-exposed group, the dissected brains were rinsed three times quickly (∼5 s) with 3 ml 70 mM KCl in AHL, alternating with 3 ml AHL, and then imaged at *t*=1, 5, 10, 20, 30 and 40 min after KCl induction. Control flies were rinsed in AHL containing no additional KCl, and imaged the same way. To quantify the GRASP fluorescence in the AL, a maximum projection of each image stack was created, and an average fluorescence intensity was calculated from an ROI encompassing the AL. Δ*f*/*f* was calculated using *t*=0 as a baseline, and the flies for each group were averaged to produce the timecourse graph. This same procedure was followed for KCl syb:GRASP induction at the olfactory PN-MB synapses (genotype *GH146-LexA/+;MB247-Gal4/Aop-syb:spGFP1–10, UAS-CD4:spGFP11*).

### Odour induction of syb:X-RASP

For these experiments, we crossed flies with the genotype *Orco-Gal4/UAS-syb:spGFP1–10* with *GH146-LexA/Aop-CD4:spGFP11* to obtain syb:GRASP between olfactory sensory neurons and GH146 projection neurons. To minimize the exposure of these flies to odours, cleaned pupae were placed individually into eppendorf tubes containing sorbitol agarose substrate (1 M sorbitol, 2% agarose). These tubes were capped with aluminium foil with pinholes to allow air flow, and the flies were placed in a well-ventilated lab space. Within 24 h of eclosion, the flies were separated into control and odour-exposed groups and placed in glass beakers with foil on top. For the odour-exposed groups, the beakers were placed in the fume hood, a small strip of filter paper was dipped into odorant (either pure or diluted in mineral oil), and placed into the beaker for 10 min. Then the paper was removed, and the beaker left open for 10 min, and then a new strip was dipped in odour, placed in the beaker, and recovered with foil. This was repeated for a total of four odour exposures. After the last exposure, flies were left in the fume hood, beaker uncovered for 1 h (or for 1, 4 and 6 h in Fig. 3f) and then dissected to isolate the brain which was then imaged in two-photon microscopy. Control flies were handled as the odour-exposed groups before dissection and imaging. Odours used: isopentyl acetate (Sigma #79857), benzyl acetate (Sigma #50475), ethyl acetate (Sigma #270989), ethyl hexanoate (Sigma #148962), geranyl acetate (Sigma #45896). To quantify the GRASP fluorescence in the AL, a maximum projection of each image stack was created (encompassing the entire AL, ∼50 μm at 1 frame per μm), and an average fluorescence intensity was calculated from an ROI encompassing the AL. To quantify GRASP fluorescence in individual glomeruli, a maximum projection of a small substack (<5 μm) was used, and the average fluorescence intensity was calculated from an ROI surrounding the glomerulus of interest. For odour experiments with two-colour X-RASP, flies of the genotype *IR84a-LexA, Aop-syb:YFP1–10/GH146-Q, QUAS-spXFP11; OR92a-Gal4, UAS-syb:spGFP1–10/+* were either grown on odour-free sorbitol food or exposed to odour pulses as above (10% S-carvone and 1% phenylacetaldehyde, diluted in mineral oil—a kind gift of T. Bozza), and both Y-RASP and G-RASP were directly visualized in two-photon microscopy using 945 nm laser light and a 525/70 filter. This set-up allowed us to simultaneously image and quantify Y- and G-RASP signals. In Fig. 6f,g monochrome images were rendered using a fire lookup table (LUT) to emphasize differences in intensity. ROIs corresponding to VL2a and VA2 were defined on projections of the two-photon stacks to measure average intensity. Unfixed fly brains were imaged using either a Prairie Ultima SGS two-photon microscope or Olympus FVMPE-RS.

### Temperature induction of syb:GRASP

Flies of the following genotype *w; Aop-syb-spGFP1–10; UAS-CD4:spGFP11/+; IR93a-LexA/VT40053-Gal4* were raised at 25 °C. One-day old adults were placed in either 15 or 35 °C incubators for 10 min. The control group was left at 25 °C. Flies were briefly anaesthetized with CO_2_, their brains were dissected in PBS, and the syb:GRASP signal at the posterior antennal lobe (PAL) was imaged immediately on a Prairie Ultima SGS two-photon microscope. In Fig. 4b–d monochrome images were rendered using a fire LUT to emphasize differences in intensity. syb:GRASP fluorescence was measured from ROIs of the cold and hot glomerulus from the maximum intensity *Z*-projection output image of the PAL.

### Light induction of syb:GRASP

R8-Tmc5 syb:GRASP flies (genotype *panR8-Gal4 /ort*^*C1a*^*-LexADBD, OK371-dVP16AD; UAS-syb:spGFP1–10, LexAop-spGFP11::CD4/+*) were used for these experiments. Before eclosion, larvae and pupae were kept on a 12 h light/dark cycle, then after eclosion, the flies were raised in constant dark or constant light (50 lx (14 W cm^−2^), white light) condition for 3–5 days. The brains were dissected, fixed in 4% PFA, and imaged using a Zeiss LSM780 confocal microscope as described in Karuppudurai *et al*.^10^. Note that, in all cases we tested (olfactory, temperature and light induction) a few days (3–5) of exposure to normal ambient stimuli is sufficient to produce adequate syb:GRASP signals for circuit mapping (which can often be easily boosted by brief application of KCl, see above).

### HEK 293 transfections

HEK293T cells (ATCC) were plated in a 24-well plate, and then each spXFP1–10 construct was co-transfected with spXFP11 to produce syb:X-RASP in *cis* using Lipofectamine 2,000. For G-RASP, *pCMV-Gal4+pUAST-syb:spGFP1–10*+*pAAV-CAG-CD4:spXFP11*; for Y-RASP, *pCMV-LexA+pLOT-syb:spYFP1–10+pAAV-CAG-CD4:spXFP11*; for C-RASP, *pCMV-QF+pQUAST-syb:spCFP1–10+pAAV-CAG-CD4:spXFP11*. Twenty-four hours after transfection, the media was removed, the cells washed briefly with D-PBS, and then new media was placed on the cells. The cells were then removed from the plate by gentle trituration, mixed together, and then plated into new wells containing poly-lysine coated coverslips. After an additional 24 h of incubation, the cells were rinsed with PBS, fixed with 4% PFA, and then imaged on an Olympus FV1200 Confocal microscope using the settings below.

### Confocal imaging of X-RASP

To separate C-RASP/G-RASP/Y-RASP from one another in the HEK cell-mixing experiment, we used spectral imaging settings on the confocal microscope. C-RASP: 458 nm laser, dichroic: 458/515, imaged 465–485 nm. G-RASP: 488 nm laser, dichroic; 405/488, imaged 500–510 nm. Y-RASP: 515 nm laser, dichroic 458/515, imaged 525–545 nm. To plot the emission spectra of X-RASP, we imaged HEK cells expressing each X-RASP in separate coverslips using a × 40 objective. We imaged using a wavelength detection range of 460–560 nm, exciting with 458, 488 and 515 nm laser lines in series, and collected images in a 5-nm band, so that we recorded a set of images for each excitation laser used. We chose 8–10 ROIs for each colour X-RASP (circling individual cells). We then normalized the fluorescence of each ROI to its maximum fluorescence value, and plotted the average normalized emission values for each X-RASP over the following windows: 460–495 nm (458 nm laser), 500–520 nm (488 nm laser) and 525–560 nm (515 nm laser).

### Two-choice assay for temperature preference behaviour

Our rapid two-choice assay for temperature preference has been described in detail before^23^. In brief, 20 3–5 days old flies are ice-anaesthetized and placed in an arena consisting of four 1 inch square, individually addressable Peltier tiles. In each trial, flies are presented for 3 min with a choice between 25 °C and a test temperature (TT) between 10 and 40 °C at 5 °C intervals. Trials are paired so that each TT is presented twice, each time in different opposing quadrants and the position of flies is recorded during each trial (by a BASLER A601FM camera) to calculate an avoidance index (AI) for each TT AI=number of flies at 25 °C − number of flies at TT)/total number of flies).

## Additional information

**How to cite this article:** Macpherson, L. J. *et al*. Dynamic labelling of neural connections in multiple colours by trans-synaptic fluorescence complementation. *Nat. Commun*. 6:10024 doi: 10.1038/ncomms10024 (2015).

## Supplementary Material

Supplementary Information Supplementary Figures 1-6, Supplementary Tables 1 and 2, and Supplementary References
