# Supplementary material for: Dynamic labelling of neural connections in multiple colours by trans-synaptic fluorescence complementation
Source: Nat Commun. 2015 Dec 4;6:10024. doi: 10.1038/ncomms10024 (PMC4686661; doi:10.1038/ncomms10024)
Supplement: Supplementary Information — Supplementary Figures 1-6, Supplementary Tables 1 and 2, and Supplementary References [file ncomms10024-s1.pdf]

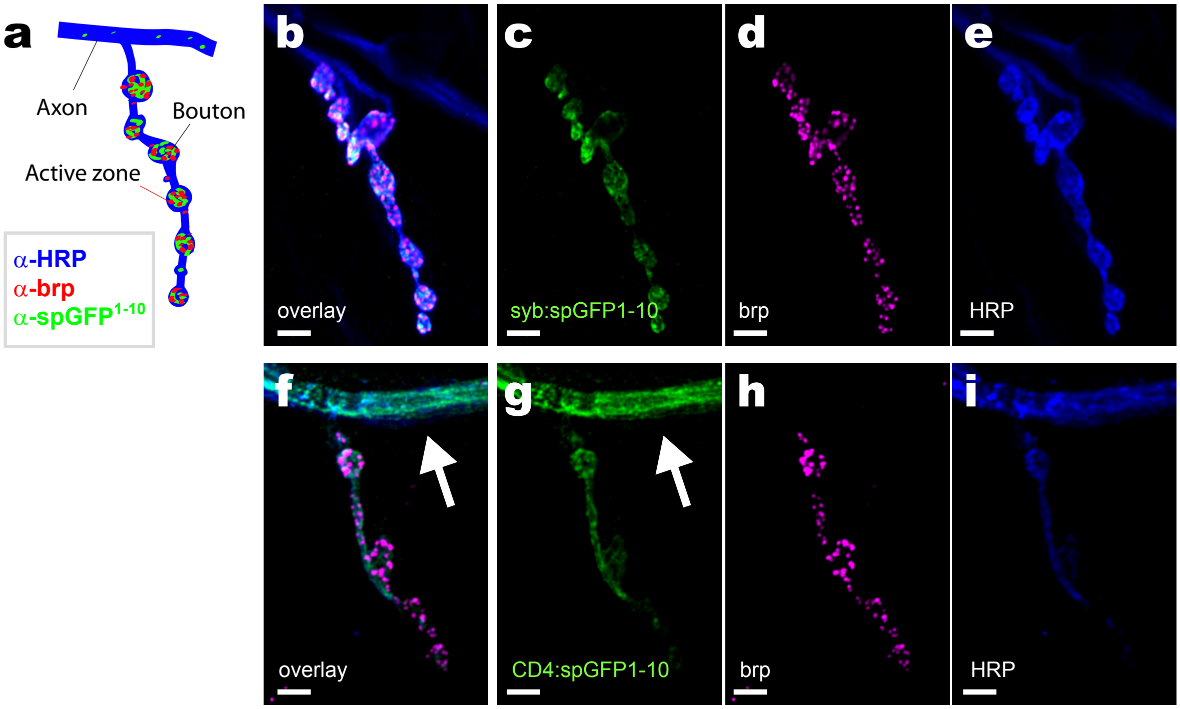

Supplementary Figure 1: syb:GFP1-10 localizes to pre-synaptic boutons in the larval neuromuscular junction. (**a**) Schematic representation of a presynaptic terminal of the larval neuromuscular junction, highlighting the antibodies used to localize spGFP1-10. Anti-HRP (blue) stains all neuronal membranes. Anti-brp (red) recognizes an active-zone marker (Bruchpilot). Anti-GFP (green) labels spGFP1-10. Here, Vglut-Gal4 was used to express UAS-syb:spGFP1-10 (**b-e**) or UAS-CD4:spGFP1-10 (**f-i**) in motor neurons innervating the larval neuromuscular junction; syb:spGFP1-10 localizes to synaptic boutons, whereas CD4:spGFP1-10 is expressed throughout the cell membrane (the arrows in f and g point to the axon). (**c,g**) Anti-GFP labeling sp-GFP1-10, green channel. (**d,h**) Anti-brp, magenta. (**e,i**) Anti-HRP, blue. Scale bars: 5 µm. Full genotypes: (**b**) VGlut-Gal4/+; UAS-syb:spGFP1-10/+; (**c**) VGlut-Gal4/UAS-CD4:spGFP1-10.

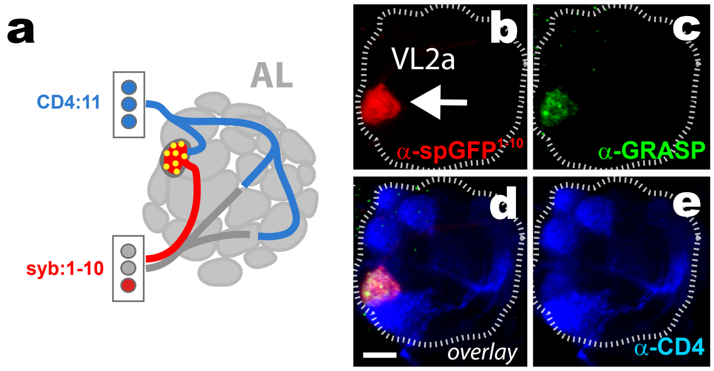

Supplementary Figure 2: Specificity of antibodies recognizing different GRASP components. (**a**) Experimental setup. (**b**) Polyclonal chicken anti-GFP antibody (abcam # 13970) preferentially labels spGFP1-10. Immunostaining using this anti-GFP labels the VL2a glomerulus brightly (red). (**c**) A monoclonal anti-GFP (Sigma #G6539) preferentially recognizes reconstituted GFP in GRASP. GRASP signal (green) is also detected in the VL2a glomerulus. (**e**) CD4:spGFP11 is expressed throughout the GH146 projection neurons and can be visualized using an anti-CD4 antibody (blue; **d** is an overlap of **b**,**c** and **e**). Full genotype: IR84a-LexA, Aop-syb:spGFP1-10 / GH146-Q , QUAS-CD4:spGFP11.

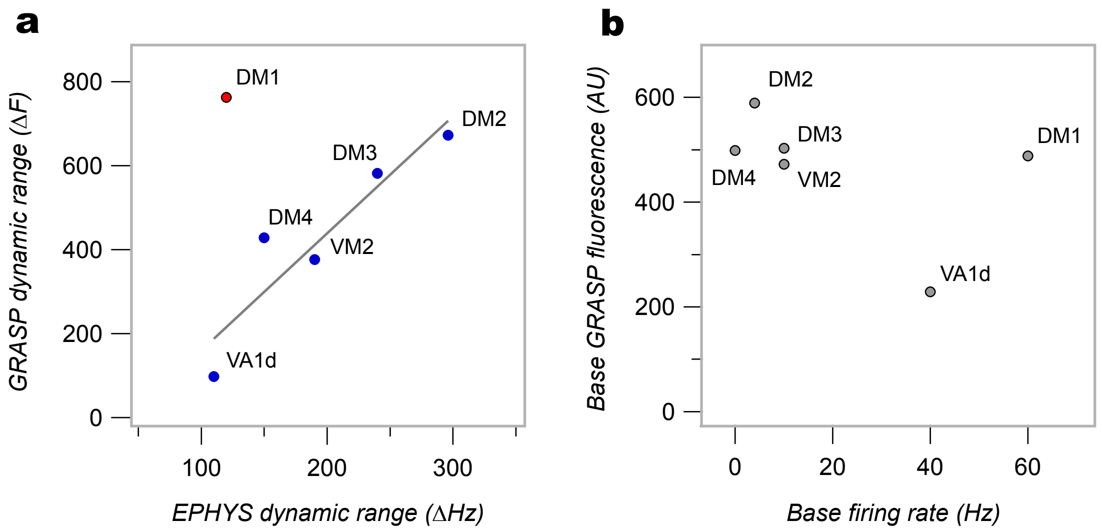

Supplementary Figure 3: The dynamic range of syb:GRASP responses for each glomerulus correlates well with the dynamic range of firing for the corresponding ORN type (as compiled from literature, see Supplementary Table 1 for all data used here). (**a**) The syb:GRASP dynamic range (calculated by subtracting the base fluorescence from the max odor-induced GRASP fluorescence, see data in Supplementary Table 1) correlates well with the dynamic range of firing frequency (similarly calculated by subtracting the baseline firing frequency from the max odor-induced response frequency; R=0.925). The DM1 glomerulus is an exception (and was excluded from regression analysis for this reason- denoted by a red dot in the plot), having a particularly large GRASP dynamic range for unclear reasons. In contrast (**b**), the baseline syb:GRASP fluorescence for each glomerulus does not obviously correlate with the basal firing rate of the corresponding ORNs. Full genotype: Orco-Gal4/GH146-LexA, Aop-CD4:spGFP11; UAS-syb:spGFP1-10/+.

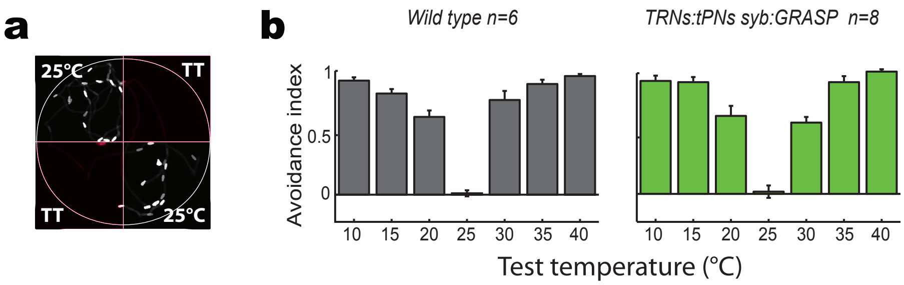

Supplementary Figure 4: Targeting syb:GRASP to the first synaptic station of the fly thermosensory system does not significantly affect the flies' temperature preference behavior in a rapid 2-choice test. (**a**) Schematic representation of the rapid 2-choice assay used to establish temperature preference: a small group of adult flies is confined to a circular arena tiled by temperature-controlled elements. Two elements are set to the flies' preferred temperature of 25°C, while the two opposing ones to a test temperature (TT, red overlay), which varies in each trial between 10 and 40°C. Trials are paired so that each TT is presented twice, each time in different opposing quadrants; an avoidance index is then calculated by computing the time spent by the flies on the different temperature quadrants (AI, see methods for details). (**b**) Flies expressing syb:GRASP at the first synaptic station of the thermosensory system (green bars) display a pattern of temperature preference that is not significantly different from that of wild-types (grey bars, right; full genotype: w ; Aop-syb-spGFP1-10; UAS-CD4:spGFP11/+ ; IR93a-LexA / VT40053-Gal4; two tailed t-tests show no significant difference between AIs for each test temperature).

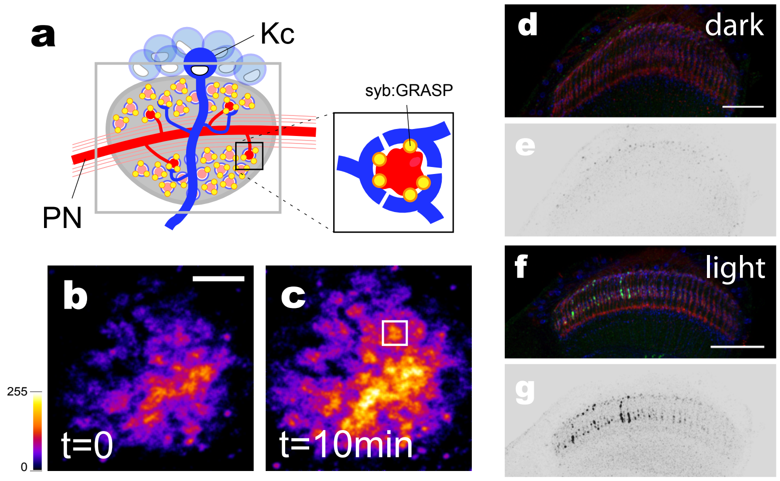

Supplementary Figure 5: Activity-dependent syb:GRASP demonstrated in the fly olfactory and visual systems. (**a**) Experimental design: olfactory PNs synapse onto Kenyon Cell dendrites in the Mushroom Body Calyx, forming microglomerular structures (PN, projection neuron; Kc, Kenyon cell; a single 'microglomerulus' is represented in the box, the Calyx is shaded in grey). (**b,c**) Syb:GRASP fluorescence detected by two-photon microscopy in the Calyx (PNs: GH146>syb:spGFP1-10, Kcs: MB247>CD4:spGFP11; the area imaged roughly corresponds to the grey box in a) is boosted by KCl treatment (by 17% $P<0.01$; One tailed t-test, $n = 6$). (**b**) and (**c**) are representative projections of the entire Calyx before and after KCl. Scale bar: 10 µm. (**d-g**) Activity-dependent syb:GRASP demonstrated in the fly visual system. For this experiment, we expressed syb:spGFP1-10 in retinal photoreceptors (R8 type, using a pan R8-Gal4 driver37, and spGFP11 in their synaptic partners Tm5c (under ortC1A-LexA control10). Flies expressing syb:GRASP at the R8:Tm5c synapse were raised either in dark (**d, e**) or in light (**f,g**) for 3-5 days after eclosion. GRASP signal (green) was observed after light exposure at the R8 terminals. Photoreceptors were labeled with 24B10 antibody. Anti-hCD4 immunolabeling (blue) outlines the morphology of Tm5c neurons (expressing CD4:spGFP11). (**e,g**) show the native green fluorescence of (**d, f**). Scale bar: 30 µm. Full genotypes: (**a-c**) GH146-LexA/+;MB247-Gal4/ Aop-syb:spGFP1-10, UAS-CD4:spGFP11; (**d-g**) panR8-Gal4 /ortC1a-LexADBD, OK371-dVP16AD; UAS-syb:spGFP1-10, Aop-CD4:spGFP11/+.

a

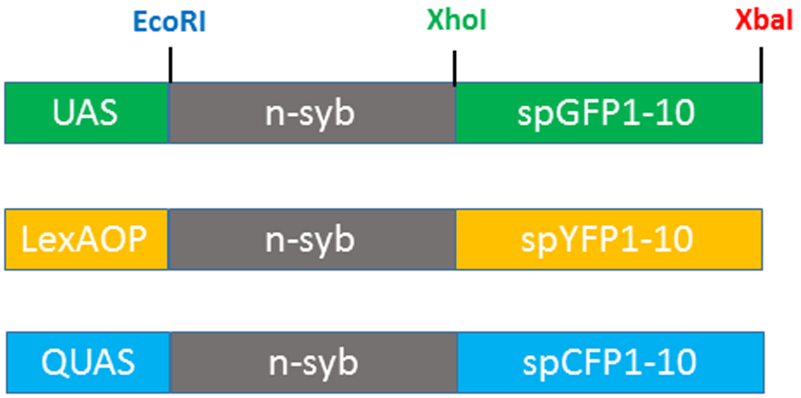

syb1-F	ACAGCG**GAATTC**GCTGAGGC
Syb2-R	TC**CTCGAG**CCACGCCGCCGTGATCGCCAG
sp10Xho-F	G**CTCGAG**GAATGTCCAAAGGAGAAGAACTGTTTACCGG
sp10Xba-R	GC**TCTAGA**CTATGTTCCTTTTTCATTTGGATCTTTGC

b

```
spGFP1-10        MSKGEELFTGVVPILVELDGDVNGHKFSVRGEGEGDATIGKLTLKFICTTGKLPVPWPTL 60
spYFP1-10        MSKGEELFTGVVPILVELDGDVNGHKFSVRGEGEGDATIGKLTLKFICTTGKLPVPWPTL 60
spCFP1-10        MSKGEELFTGVVPILVELDGDVNGHKFSVRGEGEGDATIGKLTLKFICTTGKLPVPWPTL 60
                 ************************************************************

spGFP1-10        VTTLTYGVQCFSRYPDHMKRHDFFKSAMPEGYVQERTISFKDDGKYKTRAVVKFEGDTLV 120
spYFP1-10        VTTLGYGLQCFARYPDHMKRHDFFKSAMPEGYVQERTISFKDDGKYKTRAVVKFEGDTLV 120
spCFP1-10        VTTLTWGVQCFARYPDHMKRHDFFKSAMPEGYVQERTISFKDDGKYKTRAVVKFEGDTLV 120
                 **** :*:***:************************************************

spGFP1-10        NRIELKGTDFKEDGNILGHKLEYNFNSHNVYITADKQKNGIKANFTVRHNVEDGSVQLAD 180
spYFP1-10        NRIELKGTDFKEDGNILGHKLEYNFNSHNVYITADKQKNGIKANFTVRHNVEDGSVQLAD 180
spCFP1-10        NRIELKGTDFKEDGNILGHKLEYNFISDNVYITADKQKNGIKANFTVRHNVEDGSVQLAD 180
                 ************************* *.********************************

spGFP1-10        HYQQNTPIGDGPVLLPDNHYLSTQTVLSKDPNEKGT 216
spYFP1-10        HYQQNTPIGDGPVLLPDNHYLSYQTVLSKDPNEKGT 216
spCFP1-10        HYQQNTPIGDGPVLLPDNHYLSTQTVLSKDPNEKGT 216
                 ********************** *************
```

Supplementary Figure 6: (**a**) Schematic of transgenes created for this study, and primers used for cloning. (**b**) Alignment of spGFP1-10, spYFP1-10, and spCFP1-10.

Glomerulus	Sensillum	OR	Baseline (Hz)	Max Response (Hz)	Supplementary Ref.	Av. F Background, AU	Av. Max GRASP response, AU (odor)
DM1	ab1	Or42b	60	180	[1]	488	1251 (BA 100%)
DM2	ab3A	Or22a	4	300	[2]	589	1262 (IPA 100%)
DM3	ab5B	Or47a+33b	10	250	[1]	503	1085 (IPA 100%)
DM4	ab2A	Or59b	0	150	[1]	499	927 (BA 100%)
VM2	ab8A	Or43b	10	200	[3]	472	849 (IPA 100%)
VA1d	at4	Or88a	40	150	[4]	216	326 (IPA 100%)

Supplementary Table 1: Baseline and maximum firing frequencies of ORNs ordered by the glomerulus they innervate compared to baseline and maximum syb:GRASP fluorescence recorded at each corresponding glomerulus in this study. Grey columns are a compilation of published baseline and maximum firing frequencies of ORNs ordered by the glomerulus they innervate (in Hz). The average baseline and maximum syb:GRASP fluorescence recorded in this study for each corresponding glomerulus is also tabulated (expressed in arbitrary fluorescence units or AU). All electrophysiology data comes from published sensillum recordings in which the OR expressing neuron could be unambiguously identified. 'Max response' refers to the maximum firing frequency recorded using the best activating odor. Likewise, the GRASP value for average max response refers to the best activating odor in our set. 'Av. F. Background' refers to basal fluorescence at each glomerulus in control animals kept on sugar-agar ('odorless') food, see text and methods for details. Note that this data is a subset of what is plotted in Figure 3h.

#	Name	Plasmid	P element Insertions	Average Expression (AL)
1	UAS-CD4:spGFP11	pUAST	P: 2	
2	QUAS-CD4:spGFP11	pQUAST	P: 2	
3	AOP-CD4:spGFP1-10	pLOT	P: 2	
4	UAS-syb:spGFP1-10	pUAST	P: 2	792 +/- 103
5	UAS-syb:spGFP1-10	pUAST	P: 3	981 +/- 53
6	AOP-syb:spGFP1-10	pLOT	P: 2	860 +/- 174
7	AOP-syb:spYFP1-10	pLOT	P: x	979 +/- 215
8	AOP-syb:spYFP1-10	pLOT	P: 2	920 +/- 146
9	AOP-syb:spYFP1-10	pLOT	P: 3	1081 +/- 174
10	QUAS-syb:spCFP1-10	pQUAST	P: x	905 +/- 179
12	QUAS-syb:spCFP1-10	pQUAST	P: 3	878 +/- 200
13	Or49b-QF	pBP-QF	attP18: x	

Supplementary Table 2: A list of the transgenic fly lines created for this study. The table lists the abbreviated genotype ('Name'); the plasmid backbone ('Plasmid') and chromosomal locations of each of the lines created ('P element insertions'). The last column reports average expression levels of the syb:spXFP1-10 lines, demonstrating robust expression of each transgene (n=4 flies each). To determine the average expression of *syb:spGFP1-10*, *syb:spYFP1-10*, and *syb:spCFP1-10* these transgenes were driven broadly in the AL (under the control of *Orco-Gal4*, *Orco-LexA*, and *GH146-QF*, respectively -note that we used *GH146-QF* as a *Orco-QF* line is not available). Fly brains were dissected and immunostained with Chicken anti-GFP as described (see methods), and confocal stacks were used to measure fluorescence on ROIs encompassing the entire ALs or single glomeruli, as appropriate (see methods for details; average fluorescence is reported in arbitrary units AU +/- Std Dev).

Supplementary References:

1. de Bruyne, M., K. Foster, and J.R. Carlson, *Odor coding in the Drosophila antenna.* Neuron, 2001. **30**(2): p. 537-52.

2. Pelz, D., et al., *The molecular receptive range of an olfactory receptor in vivo (Drosophila melanogaster Or22a).* J Neurobiol, 2006. **66**(14): p. 1544-63.

3. Elmore, T., et al., *Targeted mutation of a Drosophila odor receptor defines receptor requirement in a novel class of sensillum.* J Neurosci, 2003. **23**(30): p. 9906-12.

4. Dweck, H.K., et al., *Pheromones mediating copulation and attraction in Drosophila.* Proc Natl Acad Sci U S A, 2015. **112**(21): p. E2829-35.
